# Gangrenous appendicitis presenting as acute abdominal pain in a patient on automated peritoneal dialysis: a case report

**DOI:** 10.1186/1752-1947-6-309

**Published:** 2012-09-18

**Authors:** Robert Ekart, Matjaž Horvat, Miran Koželj, Breda Pečovnik Balon, Sebastjan Bevc, Radovan Hojs

**Affiliations:** 1 Department of Dialysis; Clinic for Internal Medicine, University Medical Centre Maribor, Ljubljanska 5, Maribor, SI 2000, Slovenia; 2 Department of Abdominal Surgery; Clinic for Surgery, University Medical Centre Maribor, Ljubljanska 5, Maribor, SI 2000, Slovenia; 3 Department of Nephrology; Clinic for Internal Medicine, University Medical Centre Maribor, Ljubljanska 5, Maribor, SI 2000, Slovenia

**Keywords:** Abdominal pain, Appendicitis, Myocardial infarction, Peritoneal dialysis

## Abstract

**Introduction:**

Presentations of abdominal pain in patients on peritoneal dialysis deserve maximal attention and careful differential diagnosis on admittance to medical care. In this case report a gangrenous appendicitis in a patient on automated peritoneal dialysis is presented.

**Case presentation:**

We report the case of a 38-year-old Caucasian man with end-stage renal disease who was on automated peritoneal dialysis and developed acute abdominal pain and cloudy peritoneal dialysate. Negative microbiological cultures of the peritoneal dialysis fluid and an abdominal ultrasonography misleadingly led to a diagnosis of culture negative peritonitis. It was decided to remove the peritoneal catheter but the clinical situation of the patient did not improve. An explorative laparotomy was then carried out; diffuse peritonitis and gangrenous appendicitis were found. An appendectomy was performed. Myocardial infarction and sepsis developed, and the outcome was fatal.

**Conclusion:**

A peritoneal dialysis patient with abdominal pain that persists for more than 48 hours after the usual antibiotic protocol for peritoneal dialysis-related peritonitis should immediately alert the physician to the possibility of peritonitis caused by intra-abdominal pathology. Not only peritoneal catheter removal is indicated in patients whose clinical features worsen or fail to resolve with the established intra-peritoneal antibiotic therapy but, after 72 hours, an early laparoscopy should be done and in a case of correct indication (intra-abdominal pathology) an early explorative laparotomy.

## Introduction

Acute abdominal pain could be a very serious complication in patients receiving continuous ambulatory peritoneal dialysis (CAPD) and automated peritoneal dialysis (APD). The peritonitis caused by exogenous infection related to the peritoneal catheter is a common reason for abdominal pain in these patients. Consequently, to identify whether a patient on CAPD or APD has underlying intra-abdominal pathology by means of a laparoscopy remains a diagnostic challenge for nephrologists and abdominal surgeons. A misled diagnosis may result in delayed urgent surgery and contribute to the high rate of deaths in such patients.

We present the case of a patient on APD with gangrenous appendicitis. Diagnosis was delayed and the patients`s outcome was catastrophic. According to our experience with peritoneal dialysis (PD) patients with a clinical picture of acute abdominal pain, it is necessary to think about all differential diagnostic possibilities and not only about the usual peritonitis. It is also crucial to exclude intra-abdominal pathology in those patients who do not respond promptly to intra-peritoneal antibiotics.

## Case presentation

**Figure 1:**
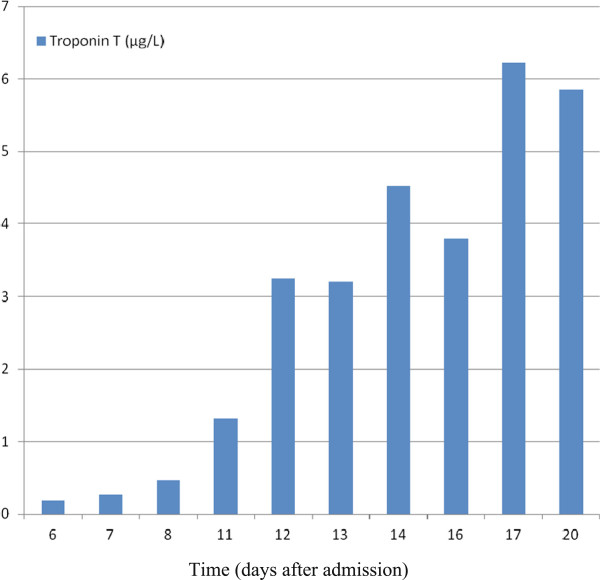
Enzymatic curve for troponin T

We present the case of a 38-year-old man with end-stage renal disease secondary to diabetes mellitus type 1 on CAPD for 18 months and on APD for 40 months. The patient, who had no previously reported episode of PD-related bacterial peritonitis, was admitted to our department with a 2-day history of emesis, cloudy peritoneal dialysate, and abdominal pain in the upper abdomen as well as diarrhea. On admittance, he was afebrile and normotensive with a blood pressure of 125/85mmHg; his pulse was regular but tachycardic at 143 beats per minute. There was no abdominal tenderness. The PD catheter exit site was clean. An electrocardiogram (ECG) showed sinus tachycardia with a rate 143 beats per minute, but no signs of myocardial ischemia were seen. The complete blood count showed white blood cells of 14.6 cells/mm^3^ with 83% segmented neutrophils, 1% unsegmented neutrophils, 7% lymphocytes, 7% monocytes, and 2% eosinophils; hematocrit 33%; and platelets of 313 × 10^3^/μL. C-reactive protein was 138mg/L and procalcitonin was 1.1ng/mL. The first dialysate leukocyte count was 1055/μL with 93% segmented neutrophils, 3% lymphocytes and 4% monocytes. The usual PD-related peritonitis was suspected and after sending the first PD effluent for culture to the laboratory, we started treatment with antibiotics according to hospital protocol: intra-peritoneal cefazoline and ceftazidime. The Gram stain of the first effluent was negative. The second Gram stain of the PD effluent 3 days after admittance was also negative. The exit site culture and coproculture were negative. The abdominal X-ray and abdominal ultrasound (US) were unrevealing. After 3 days of antibiotic therapy the clinical status of the patient did not improve. Furthermore, abdominal tenderness in the epigastrium and the upper right quadrant, inappetence and anuria, appeared. The patient was switched to hemodialysis using a central venous catheter in the vena jugularis interna. On the sixth day after admission the patient complained, for the first time, about pain in his left arm; his ECG was without signs of ischemia, and his troponin T was slightly elevated (0.19μg/L). On the seventh day after admission we decided to remove the peritoneal catheter in a surgical procedure under general anesthesia. The dialysate leukocyte count just before the operation was 4385/μL with 99% segmented neutrophils. After the surgical procedure the patient was treated with intravenous vancomycin and ceftazidime, but over the next few days his clinical situation was no better (severe abdominal pain with nausea and vomiting). On the 11th day the patient complained of pain in both arms; on the ECG signs of ischemia (T-wave inversion in inferior leads) were found. The troponin T on the same day was 1.13μg/L (Figure 1 shows the enzymatic curve) and non-ST elevation myocardial infarction was suspected. Regarding this clinical condition a cardiologist was consulted about a coronarography and an abdominal surgeon about a relaparotomy. On the same day a decision was made for surgery to be carried out. Surgeons performed an explorative laparotomy and found diffuse peritonitis and gangrenous appendicitis. An appendectomy was performed. The patient was admitted to the intensive care unit. Mechanical ventilation support was needed. The next day on an ECG ST elevation in the anterior wall was present, and troponin T was 6.22μg/L; the echocardiography showed hypokinetic anteroapical wall of the left ventricle, the ejection fraction was 40%. The patient was treated with vasopressors, intravascular antibiotics and antimycotic therapy with vancomycin, imipenem-cilastatin and fluconazole. On the 15th day of admission paralytic ileus appeared. A relaparotomy and an ileostomy were performed. After this operation the patient improved. He was extubated, lucid and talking. On the 17th day of admission the patient became worse again, he was hypotensive, in shock, and between vomiting he aspirated. On the 19th day of hospitalization bradycardia developed and cardiopulmonary resuscitation was performed. On the 20th day of admission the patient died of septic-toxic shock and multisystem failure.

## Discussion

In the medical literature 16 cases of appendicitis in adult patients on PD have been reported to date [1-11]. Most patients survived. Only one patient, a 46-year-old man, has died [1]; the reason was septic shock. Our case is the second with a fatal outcome but the first in which the patient suffered from acute myocardial infarction that influenced the postoperative clinical course and final outcome.

The presented case shows that a clinical presentation of abdominal pain in patients on PD deserves maximal attention and careful differential diagnosis on admittance to hospital. To find intra-abdominal pathology in these patients can be very difficult because the common cause of acute abdominal pain in PD patients is peritonitis caused by exogenous infection related to the peritoneal catheter. In our patient the delay in diagnosis of appendicitis was 10 days and it was consistent with the delays of between 2 and 27 days reported by others [1,2].

There are many reasons for a delay in diagnosis. The patients presented with symptoms of abdominal pain complained about diffuse pain and failed to localize the pain to a specific quadrant and thus raised the suspicion of appendicitis, perforation, diverticulitis and so on [1]. The treatment with intra-peritoneal antibiotics is indicated in PD peritonitis. Unfortunately, in PD patients with intra-abdominal pathology intra-peritoneal antibiotics probably dilute the bacterial load, retard abscess formation and also protract the course of the clinical picture [1]. The cultures of peritoneal fluid in patients with intra-abdominal pathology, especially before perforation, are often negative, and it can take several days to reveal multiple enteric Gram-negative organisms. In 13 out of 16 reported cases, the Gram stain of the peritoneal fluid was negative. In the positive cultures of peritoneal fluid mostly *Bacteroides* species and *Escherichia coli* were isolated. In our case, the patient did not complain of localized abdominal pain; the two cultures of peritoneal fluid were also negative, the second probably because of the intra-peritoneal antibiotic treatment. We also speculated that the delay in diagnosis was due to masked abdominal signs and direct local instillation of antibiotics in peritoneal fluid on the inflammatory appendix. The patient complained about pain in his arms, troponin T was elevated, and ECG changes were found. This coronary incident also had some influence on the postoperative complications, sepsis and fatal outcome.

Before the surgical procedure, two abdominal USs were performed. Unfortunately, no computed tomography (CT) scan of the abdomen was performed. The sensitivity of abdominal US and CT as diagnostic tools in appendicitis in PD patients is rather doubtful. Carmeci *et al.*[1], Yang *et al.*[3] and Yehia *et al.*[4] reported that abdominal CT scanning is not a sensitive diagnostic tool in the evaluation of these patients [1,3]. Mihout *et al.* consider that the CT scan represents a diagnostic test of choice [5]. Yehia *et al.* reported that a laparotomy was typically delayed because of negative findings on CT [4]. Carmeci *et al.* concluded in their series of six patients that the negative imaging added to the delay in diagnosis and treatment of serious intra-abdominal infections [1]. The non-localizing physical examination and negative or non-specific results of an abdominal CT scan do not rule out serious intra-abdominal disease [6]. On the basis of these different conclusions about the CT scan it can be said that a negative CT scan does not rule out an abdominal complication and should lead to further investigations by means of other procedures such as an explorative laparoscopy.

According to available data on PD patients, we can conclude that cloudy peritoneal effluent together with abdominal pain is not necessarily PD-related peritonitis. Furthermore, abdominal pain that persists for more than 48 hours after the usual antibiotic protocol for PD-related peritonitis should immediately alert the physician to the possibility of peritonitis caused by intra-abdominal pathology.

## Conclusions

To avoid the unsuccessful treatment of a PD patient with acute abdominal pain as described in this case, an intra-hospital agreement of the treatment strategy between nephrologists and abdominal surgeons was reached. In PD patients whose clinical features worsen or fail to resolve with the established intra-peritoneal antibiotic therapy not only peritoneal catheter removal is indicated but a laparoscopy should be done after 72 hours, and in the case of correct indication (intra-abdominal pathology) an early explorative laparotomy.

## Consent

Written informed consent was obtained from the patient´s next-of-kin for the publication of this case report. A copy of the written consent is available for review by the Editor-in-Chief of this journal.

## Competing interests

The authors declare that they have no competing interests.

## Authors’ contributions

RE, MH, MK, BPB and SB were the treating physicians of the patient reported. The manuscript was prepared by RE and RH. All authors read and approved the final version.
